# Spatial–temporal transmission dynamics of HIV-1 CRF01_AE in Indonesia

**DOI:** 10.1038/s41598-024-59820-y

**Published:** 2024-05-10

**Authors:** Siti Qamariyah Khairunisa, Dwi Wahyu Indriati, Ni Luh Ayu Megasari, Shuhei Ueda, Tomohiro Kotaki, Muhamad Fahmi, Masahiro Ito, Brian Eka Rachman, Afif Nurul Hidayati, Nasronudin, Masanori Kameoka

**Affiliations:** 1 Faculty of Medicine, Universitas Airlangga, Surabaya, Indonesia; 2 Indonesian-Japan Collaborative Research Center for Emerging and Re-Emerging Infectious Diseases, Institute of Tropical Disease, Universitas Airlangga, Surabaya, Indonesia; 3 Department of Health, Vocational Faculty, Universitas Airlangga, Surabaya, Indonesia; 4 Postgraduate School, Universitas Airlangga, Surabaya, Indonesia; 5 Center for Infectious Diseases, Kobe University Graduate School of Medicine, Hyogo, Japan; 6 Department of Public Health, Kobe University Graduate School of Health Sciences, 7-10-2 Tomogaoka, Suma-ku, Kobe, Hyogo 654-0142 Japan; 7 Department of Virology, Research Institute for Microbial Diseases, Osaka University, Osaka, Japan; 8 Research Department, Research Institute for Humanity and Nature, Kyoto, Japan; 9 Research Organization of Science and Technology, Ritsumeikan University, Kusatsu, Japan; 10 Department of Bioinformatics, College of Life Sciences, Ritsumeikan University, Kusatsu, Japan

**Keywords:** HIV-1, CRF01_AE, Spatial–temporal, Indonesia, Retrovirus, Viral epidemiology

## Abstract

Human immunodeficiency virus type 1 (HIV-1) remains a serious health threat in Indonesia. In particular, the CRF01_AE viruses were the predominant HIV-1 strains in various cities in Indonesia. However, information on the dynamic transmission characteristics and spatial–temporal transmission of HIV-1 CRF01_AE in Indonesia is limited. Therefore, the present study examined the spatial–temporal transmission networks and evolutionary characteristics of HIV-1 CRF01_AE in Indonesia. To clarify the epidemiological connection between CRF01_AE outbreaks in Indonesia and the rest of the world, we performed phylogenetic studies on nearly full genomes of CRF01_AE viruses isolated in Indonesia. Our results showed that five epidemic clades, namely, IDN clades 1–5, of CRF01_AE were found in Indonesia. To determine the potential source and mode of transmission of CRF01_AE, we performed Bayesian analysis and built maximum clade credibility trees for each clade. Our study revealed that CRF01_AE viruses were commonly introduced into Indonesia from Southeast Asia, particularly Thailand. The CRF01_AE viruses might have spread through major pandemics in Asian countries, such as China, Vietnam, and Laos, rather than being introduced directly from Africa in the early 1980s. This study has major implications for public health practice and policy development in Indonesia. The contributions of this study include understanding the dynamics of HIV-1 transmission that is important for the implementation of HIV disease control and prevention strategies in Indonesia.

## Introduction

Human immunodeficiency virus (HIV) attacks the human body’s immune system. HIV infection has become a major global public health issue due to its increasing number of cases yearly. Since the first HIV case was reported in 1987 in Bali, Indonesia, it is estimated that HIV cases have spread to 386 cities throughout numerous provinces in Indonesia^1^. The Joint United Nations Program on HIV/acquired immunodeficiency syndrome (AIDS) (UNAIDS) estimates that the number of adults and children newly infected with HIV worldwide at the end of 2022 had decreased by 59% since the peak in 1995. Approximately 1.3 million (1–1.7 million) people were newly infected with HIV, compared to 3.2 million (2.5–4.3 million) people in 1995. However, people living with HIV with access to antiretroviral therapy (ART) were only 76% (65–89%)^2^. Meanwhile, the new HIV infections in Indonesia by the end of 2022 have been reduced by 52% since 2010. However, AIDS-related deaths have increased by 60% since 2010, and the incidence–mortality ratio also increased with only 33% coverage of ART among Indonesians living with HIV/AIDS. Thus, HIV/AIDS has evidently become a serious health problem in this country^3^, and Indonesia has the largest number of HIV infection cases in Southeast Asia. There are three mechanisms of HIV transmission: (1) vertical transmission from HIV-infected mother to child (during pregnancy, delivery, and breast-feeding), (2) sexual transmission (homosexual or heterosexual), and (3) horizontal transmission through blood transfusion or contaminated blood transfusion (e.g., sharing injection material, tattoo, piercing, blood transfusion, organ transplantation, hemodialysis, and dental care)^4,5^.

Generally, recent HIV infection cases in Indonesia have been dominated by horizontal transmission through sexual intercourse, which comprises 89% of the total cases^6^. HIV cases were generally concentrated among key affected populations due to their high-risk sexual intercourse behavior. The key affected population was commonly female sex workers, male who have sex with male, transgenders, and people who inject drugs. Among these key populations, HIV-infected prevalence reach 30%, which is 100 times higher compared to HIV-infected prevalence in general adult residents (0.3%)^7^.

HIV has a high mutation rate, leading to a high level of genetic variation. Currently, HIV is classified into two types: HIV type 1 (HIV-1) and HIV type 2 (HIV-2). HIV-1 is classified into groups M (main), O (outline), N (new or nonM/nonO), and P (pending). Among them, group M viruses cause a pandemic. Group M is further divided into nine subtypes (A, B, C, D, F, G, H, J, and K) and several circulating recombinant forms (CRFs) generated by the intersubtypes and genomic recombination of CRFs. The global distribution of HIV-1 subtypes and CRFs is possibly affected by several factors, such as migration, cultural changes, social and political factors, international trades, and human genetics^8^.

The global spread of multiple, genetically diverse viruses was characterized by a geographical dispersion^9,10^. Subtype B became prevalent in practically all sections of Europe and the Americas^9^, while a range of subtypes and intersubtype recombinants are found with the great diversity in African continent^11–13^. Meanwhile, CRF01_AE, one of the most widespread CRFs in the world, has spread throughout Southeast Asia, including Thailand, the Philippines, Singapore, and Vietnam^14,15^. CRF01_AE is the earliest identified CRF in Thailand and has spread over Southeast Asian countries^16^. It is the dominant HIV-1 CRF in Southeast Asia and other Asian countries, but information regarding the molecular dynamics of CRF01_AE is still limited. Some research has reported that CRF01_AE is the most dominant HIV-1 CRF in Indonesia^17,18^. Monitoring the molecular dynamics of CRF01_AE HIV-1 using the phylogenetic approach was especially necessary to acknowledge characteristic evolution, spatial transmission, and temporal transmission of HIV-1 in the region^19^.

The molecular dynamics of HIV-1 is not only about identifying the molecular dynamic epidemiology, but it also involves identifying its influence in providing suitable therapy and vaccine^19^. HIV-1 subtypes and CRFs exhibit a certain level of genetic diversity. Mutations and polymorphisms are identified in the amino acid sequences of viral enzymes encoded in the *pol* region, which could impact responses to antiretroviral therapy^19^. In addition, the different immunological responses consider the need for a suitable therapy given according to the infected HIV-1 subtype or CRF^20^. In addition to therapy, HIV-1 genetics has emerged as the main challenge in developing a vaccine. Mutations that arise due to the HIV-1 evolution might be changing virus characteristics, including pathogenesis and antigenesis. For instance, amino acid substitutions in the 3rd variable loop (V3) region in envelope glycoprotein gp120 affect viral cell tropism^21^ , while mutations accumulated in the gp120 during the course of infection affect the antigenicity^22^. Therefore, developing a vaccine based on the region’s most prevalent HIV-1 subtype or CRF may be more advantageous for eliciting an adequate immune response corresponding to the strain that will most likely cause infection^5,19^.

As a country with a high incidence of HIV-1 infection in Southeast Asia^23^, Indonesia may contribute to the spread of HIV to other Asian countries, particularly in the context of CRF01_AE viruses. Concern about its potential contribution has raised significantly. However, information on the evolution of dynamic transmission characteristic, spatial transmission, and temporal HIV-1 CRF01_AE in Indonesia is lacking. The lack of such information poses a significant challenge in effectively combating the HIV epidemic. Therefore, this study uses the phylogenetic approach to analyze the characteristic evolution, spatial transmission, and temporal transmission of HIV-1 CRF01_AE in Indonesia. This analysis was performed on sequences of nearly full-length HIV-1 CRF01_AE genomes obtained from HIV-1-infected individuals in multiple regions of Indonesia. This study can provide valuable information that is not only important for Indonesia but also has broader implications for controlling and preventing HIV-1 transmission in the wider Asian region.

## Results

### Study population

Molecular epidemiological studies were carried out between 2013 and 2020 in several regions in Indonesia^18,24–32^. These studies aimed to identify HIV-1 subtypes and CRFs that circulate in Indonesia by sequencing the *pol* genes encoding reverse transcriptase and protease, as well as *gag* and *env* genes. A total of 478 samples were successfully sequenced: 70% showed genomic fragments of CRF01_AE, whereas 30% were non-CRF01_AE. Of the non-CRF01_AE genomic fragments, 30% contained 10% recombinant viruses with CRF01_AE and subtype B fragments; 2% CRF02_AG; 12% subtype B; and 6% unique recombinant viruses^18,24–30,33,34^. Twenty strains of CRF01_AE were confirmed using nearly full-length genomic sequences. Based on 20 data scenarios, the average productive age is 34 years and is dominated by 55% of the male population with a 65% marriage rate (Table 1). In this study, heterosexual transmission (85%) was predominant, and 25% of participants had received ART (Table 1).

**Table 1:** Demographic characteristics of infected individuals with 20 CRF01_AE strains in Indonesia.

ID sample	Region of origin	Age	Sex	Status	Risk factor	Treatment	Sampling year
MN34	Manado	40	Male	Single	Homosexual intercourse	Drug treated	2016
MN44	Manado	33	Female	Married	Heterosexual intercourse	Drug treated	2016
MER UM6	Manado	35	Male	Married	Heterosexual intercourse	Drug naïve	2018
HIP10	Papua	32	Female	Married	Heterosexual intercourse	Drug treated	2014
HIP6	Papua	28	Female	Married	Heterosexual intercourse	Drug naïve	2014
CEN5	Papua	40	Male	Married	Heterosexual intercourse	Drug naïve	2018
PB15	Sorong	32	Female	Married	Heterosexual intercourse	Drug naïve	2014
PB16	Sorong	41	Male	Single	Heterosexual intercourse	Drug naïve	2014
PB3	Sorong	26	Female	Married	Heterosexual intercourse	Drug naïve	2014
PB24	Sorong	48	Male	Single	Heterosexual intercourse	Drug naïve	2014
IDU10	Surabaya	30	Male	Married	Intravenous drug use	Drug naïve	2012
PJ68	Surabaya	25	Female	Single	Heterosexual intercourse	Drug naïve	2012
PJ28	Surabaya	41	Female	Single	Heterosexual intercourse	Drug naïve	2012
SBY2-19	Surabaya	36	Male	Married	Heterosexual intercourse	Drug naïve	2019
SM44	Surabaya	25	Female	Single	Heterosexual intercourse	Drug naïve	2012
BR2	Pontianak	33	Female	Married	Heterosexual intercourse	Drug naïve	2014
BR4	Pontianak	32	Male	Married	Intravenous drug use	Drug naïve	2014
SS40	Jakarta	35	Male	Married	Heterosexual intercourse	Drug naïve	2014
H44	Bali	29	Male	Single	Heterosexual intercourse	Drug treated	2016
H41	Bali	42	Male	Married	Heterosexual intercourse	Drug treated	2016

## Classification of the Indonesian HIV-1 CRF01_AE viruses into five clades

The genetic variability of HIV-1, reflected in subtypes and recombinant forms, affects the rate of progression, virus tropism, and drug resistance patterns^10^. Therefore, reconnoitering subtype diversity across populations provides valuable information about virus spread^11^. The current study aims to present molecular surveillance data on HIV variant evolution in recent years, including the transmission route of HIV-1 CRF01_AE strains in Indonesia and the rest of the world. Phylogenetic analysis was performed using 20 nearly full-length genomes of HIV-1 CRF01_AE viruses from Indonesia. Moreover, 913 corresponding sequences of HIV-1 CRF01_AE viruses from various countries that are available in the Los Alamos National Laboratory HIV Sequence Database (LANL) were used to study the epidemiology of HIV-1 CRF01_AE in Indonesia and its relationship to the global epidemic. In this study, 20 nearly complete genomic sequences of HIV-1 CRF01_AE were obtained from various regions of Indonesia, including Surabaya, Jakarta, Bali, Pontianak, Manado, Papua, and West Papua. Of the 913 sequences, most were obtained from Thailand, China (including Hong Kong), Vietnam, Japan, Laos, and the Philippines, and other Asian and global countries, such as Myanmar, Iran, the Central African Republic, Afghanistan, USA, Sweden, Cameroon, London, Bulgaria, and Slovenia. A sequence previously obtained from an Indonesian patient was included in 913 sequences retrieved from LANL. This phylogenetic tree was correlated with three subtype C lines that were outliers in the analysis.

**Figure 1:**
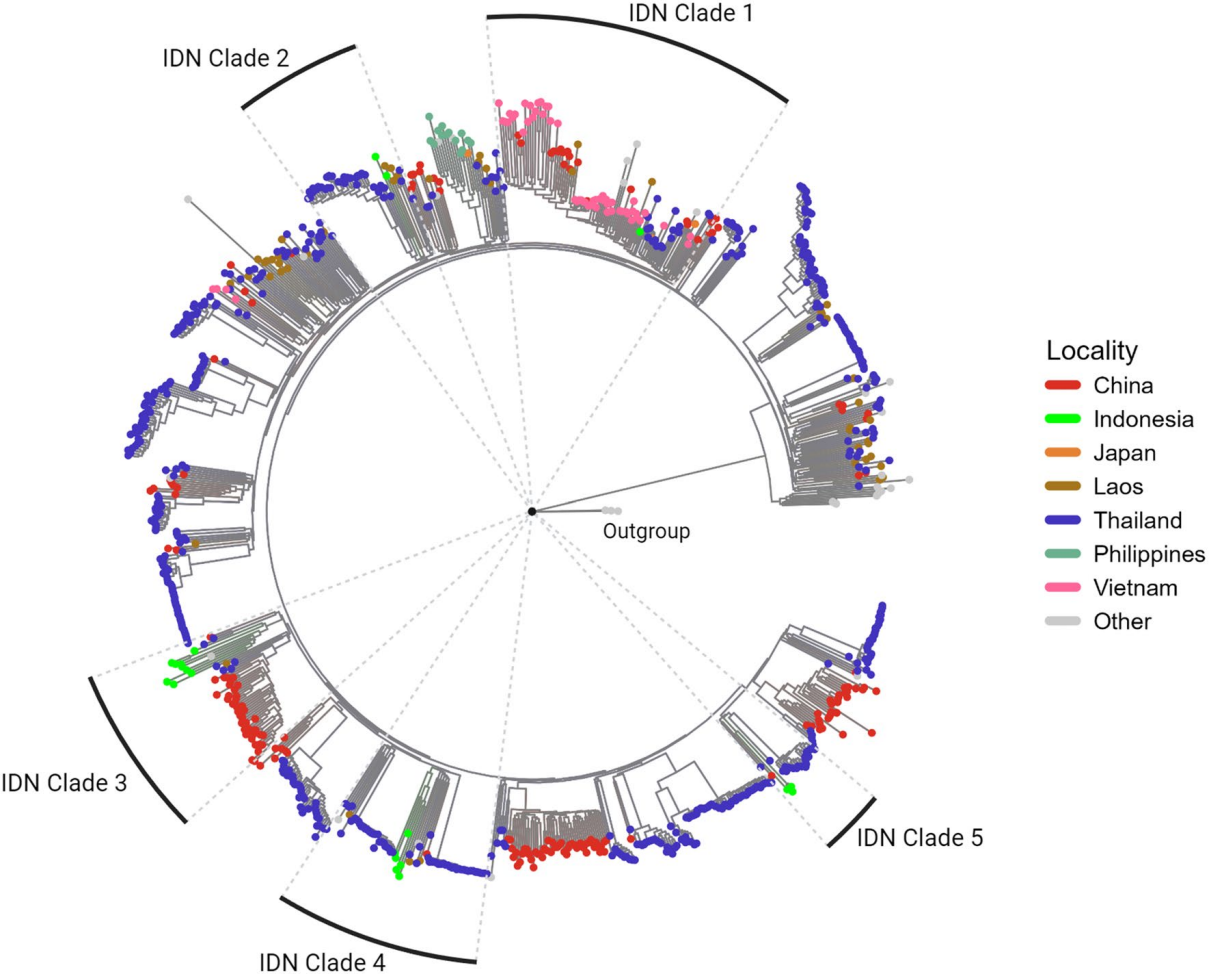
Comparative phylogenetic analysis of global CRF01_AE strains and Indonesian specimens: To investigate potential linkages between CRF01_AE strains in Indonesia and the rest of the world, we conducted a phylogenetic analysis using selected genome sequences obtained through an online BLAST search and comparing them to our Indonesia-collected CRF01_AE strains. The maximum likelihood tree was constructed using genome sequences under the GTR + F + R10 model, which was chosen as the best-fit model based on the Bayesian Information Criterion. A distinct color represents the country of origin of each sequence (on the tree). Different colors were used for sequences from China (CN), Indonesia (IDN), Japan (JP), Laos (LA), Thailand (TH), the Philippines (PH), Vietnam (VN), and all other countries combined. The ML tree is rooted with four subtype C sequences that originated in Zambia, India, South Africa, and Ethiopia, serving as the outgroup. This analysis provides insight into the possible relationships between CRF01_AE strains in Indonesia and those found worldwide. This figure was created with Biorender (http://biorender.com/ ).

**Figure 2:**
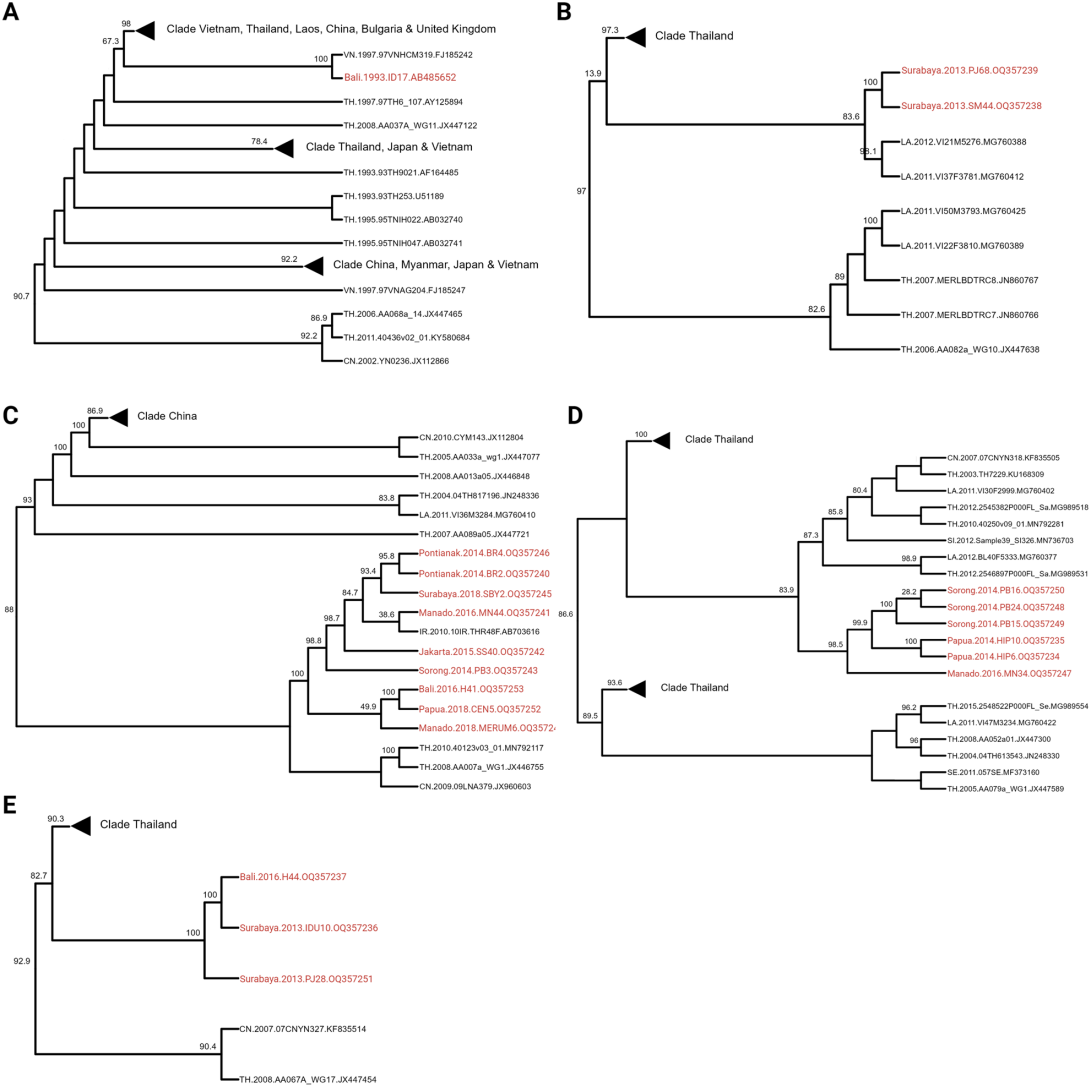
Phylogenetic tree of each clade of global CRF01_AE strains in Indonesia, (**A**) IDN Clade 1, (**B**) IDN Clade 2, (**C**) IDN Clade 3, (**D**) IDN Clade 4, and (**E**) IDN Clade 5. This figure was created using Biorender (http://biorender.com/ ).

Based on the phylogenetic analysis, the HIV-1 CRF01_AE strains in Indonesia, including sequences obtained in this study and that previously reported were classified into five distinct clades: IDN clades 1, 2, 3, 4, and 5 (Fig. 1). The clades were defined by inspecting the topology of the phylogenetic tree and branch support, with genetic distance data revealing overlaps within and between clades (Supplementary Table S1); thus, precluding genetic distance use as a sole criterion for clade classification. This underscores the complexity of viral genetic relationships of HIV-1 CRF01_AE. Figure 2 shows detailed phylogenetic analysis of each clade. Twenty Indonesian samples were analyzed in this study, of which 100% belonged to the Thailand clade. Local transmission of a few viruses from China, Vietnam, and Laos caused the CRF01_AE epidemic in Indonesia. In contrast, the Thai sequences are dispersed throughout the phylogenetic tree and are located at the base of many monophyletic clusters, thus indicating extensive interactions between Chinese, Vietnam, Laos, and Thai strains. The Indonesian strains in the Thailand clade are similar to those from China, Vietnam, Laos, and Iran.

## Spatial–temporal scale of HIV-1 CRF01_AE strains in Indonesia

**Figure 3:**
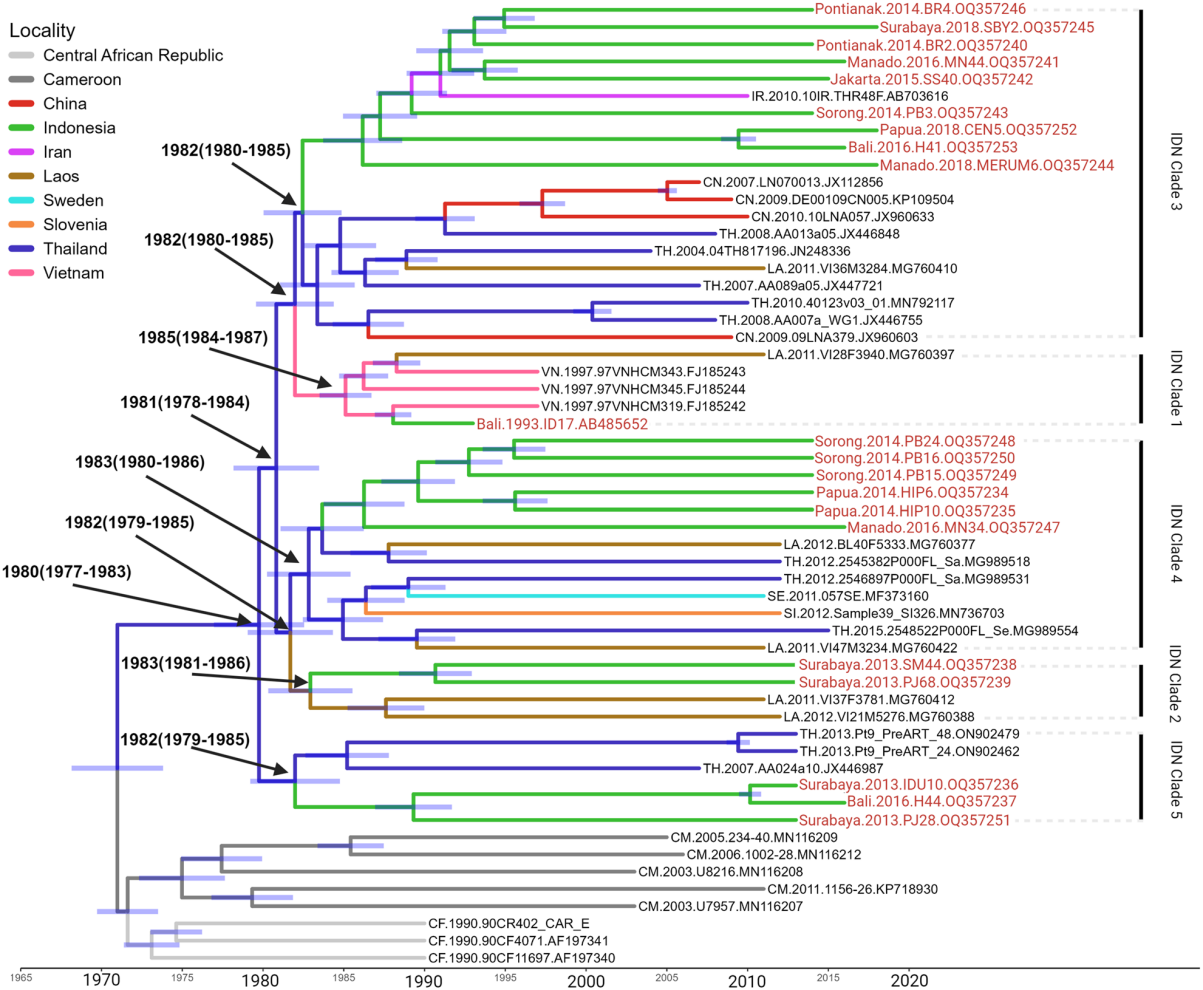
The origins and dispersal history of HIV-1 CRF01_AE, which is associated with viral transmission in Indonesia. The maximum clade credibility (MCC) tree was constructed using a subset of sequences (n = 52) that exhibit a high degree of relatedness to Indonesian samples based on the ML tree. The analysis was performed under the HKY + G sites substitution model, the strict clock model, and the coalescence: constant size model. The branch colors indicate the estimated ancestral locations of clades and the sampling locations for each sequence. This visualization reveals the origins and dispersal history of HIV-1 CRF01_AE strains associated with viral transmission in Indonesia. The tMRCA means and 95% credible intervals (in parentheses) for the key nodes are indicated. The sequences’ geographic origins are color-coded, and two-letter country codes were meant as follows: CM, Cameroon; CF, Central African Republic; IR, Iran; LA, Laos; VN, Vietnam; TH, Thailand; CN, China; Sl, Slovenia; SE, Sweden. This figure was created with Biorender (http://biorender.com/ ).

To identify the potential origin and transmission route of Indonesian HIV-1 CRF01_AE strains, we constructed maximum clade credibility (MCC) trees of five clades that included samples from Indonesia, and those related to such samples or have a unique location of origin are selected to minimize redundancy (Fig. 3). This time, we used 20 sequences of nearly full-length HIV-1 CRF01_AE from Indonesia to construct the phylogenetic tree. According to the calculation of the posterior probability that includes estimated times of the most recent common ancestors (tMRCAs), we estimate that these HIV-1 CRF01_AE strains were introduced into Indonesia as early as 1980 [95% credible interval (CI): 1977–1983] according to the most recent common ancestor of the clades. Figure 3 depicts that Indonesian samples were mainly concentrated on IDN clades 3 and 4. IDN clade 1 originates from Bali (ID17, AB485652) and is closely related to strains found in Vietnam (VN) and Laos (LA). The Bali sample were reported in 1993. Since 1985, it has been introduced and spread throughout Indonesia (CI: 1984–1987). IDN clade 2 originates from Surabaya (PJ68, OQ357239 and SM44, OQ357238) and shows similarities with Laos strains. Since 1983 (CI: 1981–1986), it has been introduced and spread throughout Indonesia. Meanwhile, IDN clade 3 is composed of Indonesian specimens originating from Sorong (PB3, GenBank accession number OQ357243), Papua (CEN5, OQ357252), Pontianak (BR4, OQ357246 and BR2, OQ357240), Surabaya (SBY2, OQ357245), Jakarta (SS40, OQ357242), Bali (H41, OQ357253), and Manado (MN44, OQ357241 and MERUM6, OQ357244). It showed similarities to HIV-1 strains detected in Thailand (TH), China (CN), Laos (LA), and Iran (IR). IDN clade 3 was introduced and spread to Indonesia since 1982 (CI: 1980–1985). IDN clade 4 consists of Indonesian samples originating from Sorong (PB16, OQ357250; PB24, OQ357248; and PB15, OQ357249), Papua (HIP10, OQ357235 and HIP6, OQ357234), and Manado (MN34, OQ357247). It showed similarities to the Thai and Laos strains and was introduced and spread to Indonesia in 1983 (CI: 1980–1986). Lastly, IDN Clade 5 is composed of Indonesian samples from Surabaya and Bali and showed similarity with the Thai strains. This was introduced and spread to Indonesia since 1982 (CI: 1979–1985).

## Discussion

Here, using 20 nearly full-length CRF01_AE genomic sequences collected in Indonesia between 2013 and 2020, we investigated the transmission network and spatial–temporal dynamics of the HIV-1 CRF01_AE epidemic. Studies on the distribution of HIV-1 subtypes and CRFs in various cities in Indonesia revealed that CRF01_AE was a major epidemic HIV-1 CRF in Medan, Riau Islands, Jakarta, Surabaya, Pontianak, Makasar, Manado, Bali, and Maumere^26^. CRF01_AE is also known to be dominant in Southeast Asian countries including Thailand, the Philippines, Singapore, and Vietnam, as well as in East Asian countries, such as Japan, China (including Hong Kong and Taiwan), and South Korea^35^. In addition, CRF01_AE is reported to have now become part of the global HIV-1 epidemic, as reported in North America, Europe, Central and West Africa, Asia, and Australia^36^.

We particularly focused on CRF01_AE, one of the main HIV-1 strains in Indonesia, to provide a deeper understanding of the dynamics of these epidemics within the country and their evolving trends. Results of phylogenetic tree analyses using nearly full-length CRF01_AE genomic sequences reveal that the CRF01_AE strain originating from Thailand predominates the spread pattern of this strain within Indonesia. Thailand is the epicenter of the HIV-1 CRF01_AE epidemic in the world^37^. The Thai CRF01_AE strain has played an important role as the main spreader in the global HIV-1 CRF01_AE epidemic (to approximately 17–20 European countries). The UK, Slovenia, Switzerland, and Austria are among the countries with the highest import transmission ratios of Thailand’s CRF01_AE strain^38^.

**Figure 4:**
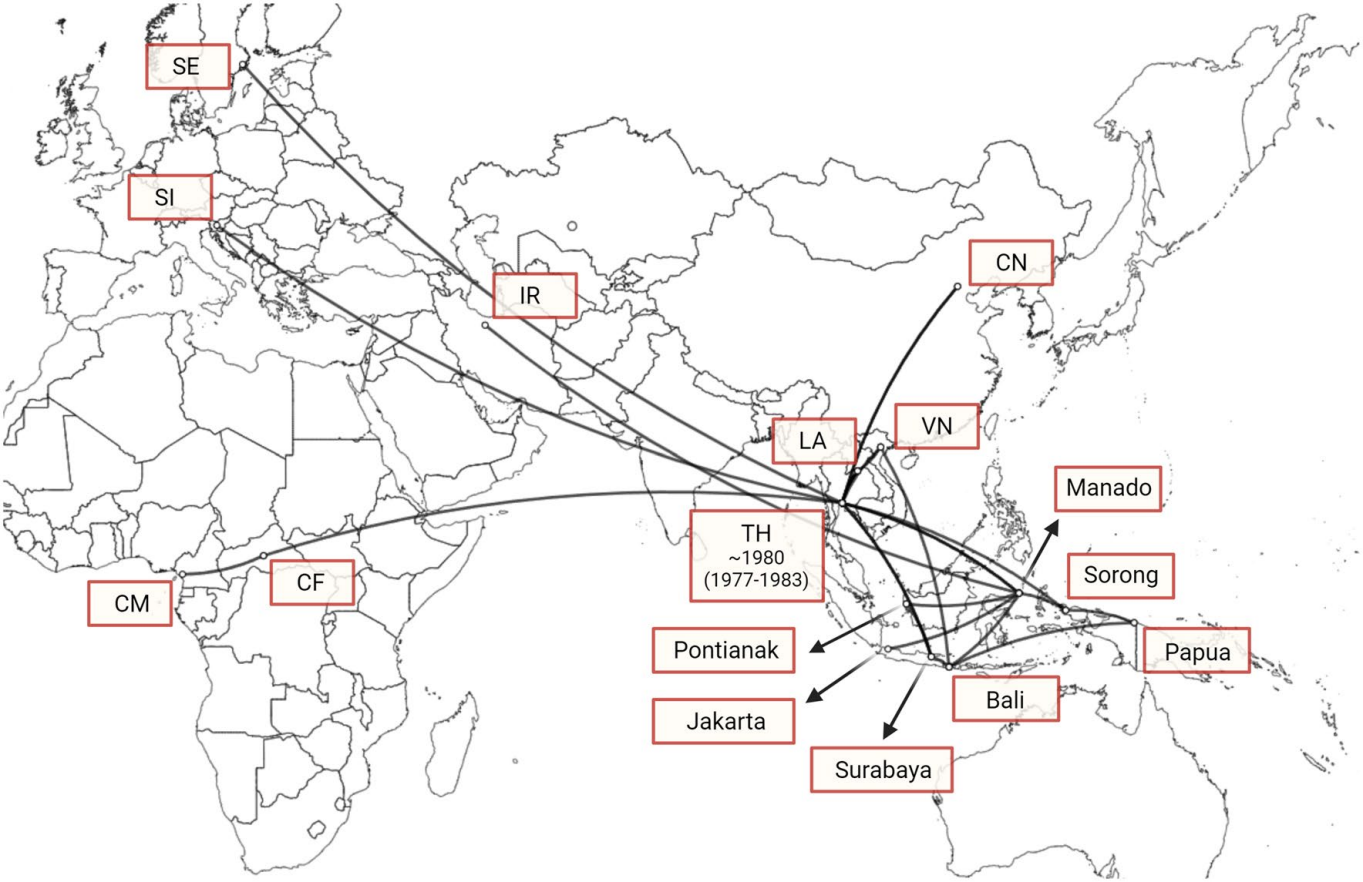
Estimated global dispersal of HIV-1 CRF01_AE strains associated with transmission in Indonesia. This phylogeography, which depicts the spatial distribution of the virus, was generated from the MCC tree using SpreaD3. Two-letter country codes were meant as follows: CM, Cameroon; CF, Central African Republic; IR, Iran; LA, Laos; VN, Vietnam; TH, Thailand; CN, China; Sl, Slovenia; SE, Sweden. This figure was created using Biorender (http://biorender.com/ ).

The spread of CRF01_AE strains is caused by several factors, including human migration, tourism, and trade. Thailand is a tourist destination for travelers from England, France, Italy, Switzerland, Russia, Denmark, and Finland. In addition to tourism, Japan’s trade relations with numerous Asian, European, and American countries have contributed to the spread of CRF01_AE^38^. Apart from Thailand, China, Vietnam, and Laos also contributed to the spread of the HIV-1 CRF01_AE strain. This indicates that CRF01_AE is endemic to these countries resulting from several virus introductions, which then spread locally. As shown in Fig. 4, the spatial and temporal spread of the HIV-1 CRF01_AE virus in Indonesia can occur in five distinct ways. CRF01_AE initially emerged in Africa but began migrating to Thailand and its neighboring countries in 1977 (Fig. 3). The transmission of CRF01_AE from Thailand to Indonesia began in the 1980s (Fig. 3). Although CRF01_AE predominantly originated from Thailand, other subtypes from Laos and Vietnam also contributed to the diversity of CRF01_AE in Indonesia. CRF01_AE spread to Southeast Asia and was discovered for the first time in Thailand in 1985 (IC: 1984–1987). These data revealed four important findings regarding the transmission of HIV-1 CRF01_AE. First, the CRF01_AE virus spread to Vietnam, Laos, China, Sweden, and Slovenia. Second, sexual transmission is the main source of CRF01_AE virus transmission (Table 1), and the CRF01_AE viruses were introduced to Indonesia via Thailand in 1980 (IC: 1977–1983). Third, Thai CRF01_AE strains spread to Vietnam and were then introduced to Indonesia in 1985 (IC: 1984–1987). Fourth, Thai CRF01_AE strains spread to Laos, then introduced to Indonesia in 1983 (IC: 1981–1986), and exported from Indonesia to Iran.

The limitations of the present study are as follows: Firstly, while we successfully amplified the nearly full-length genomic sequences of CRF01_AE viruses using primers reported in previous publications, we could not ascertain their efficacy for more recently evolved CRF01_AE viral genomes. Nonetheless, we believe these primers were designed to hybridize consensus sequences in the viral genome, and potentially serving as universal primers for amplifying nearly full-length genomic sequences of currently circulating HIV-1 CRF01_AE viruses in Indonesia. Secondly, only one nearly full-length sequence of the Indonesian CRF01_AE strain (GenBank accession no.: AB485652) has been registered in the HIV database besides our registration. In addition, we obtained only 20 sequences in the present study, partly due to budget constraints and sample quality issues. Consequently, the information on CRF01_AE genomic sequences in Indonesia remains quite limited. While several CRF01_AE genes have been registered from various countries, Indonesia has contributed only a limited number of sequences. Therefore, the analysis of viral transmission routes may be biased. Thirdly, our sample collection did not cover all geographic regions of Indonesia. Consequently, our data can only be applied to regions/cities where we obtained CRF01_AE genomic sequences. Thus, our data may not accurately represent the transmission routes of Indonesian CRF01_AE viruses. These limitations underscore the need for caution in interpreting our findings. However, we believe our data is crucial for gaining a deeper understanding of the spatial–temporal transmission dynamics of Indonesian CRF01_AE viruses.

## Conclusion

CRF01_AE is an HIV-1 CRF that dominates and is prevalent in Southeast Asia; it was discovered for the first time in Thailand in 1985. Sexual transmission is the principal method by which this CRF spreads from Thailand to other countries. The CRF01_AE virus was then introduced to Indonesia via Thailand in 1980, before spreading to Vietnam and reaching Indonesia in 1985 (CI: 1984–1987). Then, it spread from Thailand to Laos and entered Indonesia in 1983 (CI: 1981–1986). Moreover, this CRF was exported from Indonesia to Iran. The HIV-1 CRF01_AE strain has been distributed to Indonesia through various origin routes, including Thailand, China, Laos, and Vietnam. Several risk factors contribute to the spread of the CRF01_AE strain in Indonesia, including international trade, tourism (especially in Bali, a popular tourist destination), human migration, and good transit routes.

## Methods

### Nearly full genome sequencing

We conducted a nearly full genomic sequence of Indonesian HIV-1 CRF01_AE viruses. Cellular DNA was extracted from peripheral blood samples of HIV-1-infected individuals collected at several different regions in Indonesia using the QIAamp DNA blood mini kit (Qiagen, Hilden, Germany), as described previously^17,25^. The nearly full-length HIV-1 CRF01_AE genome was subsequently amplified in two fragments. Polymerase chain reaction (PCR) was performed using the Expand Long Template PCR System (Roche, Basel, Swiss). Regarding the former fragment, first-round nested PCR was conducted with the primers msf12b^39,40^ and DRIN02^41^, followed by second-round nested PCR with primers f2nst^39^ and DRIN04^41^. The following PCR conditions were utilized: In the first PCR, the initial denaturation temperature was 94 °C for 2 min, followed by 10 cycles of 94 °C for 10 s, 55 °C for 30 s, and 68 °C for 4 min; 25 cycles of 94 °C for 15 s, 55 °C for 30 s, and 68 °C for 4 min and 20 s; and the final extension at 68 °C for 7 min. In subsequent cycles, elongation times were increased by 20 s per cycle. In the second PCR, 50 °C was used as the annealing temperature. Regarding the latter fragment, first-round PCR was performed with primers DRIN01^41^ and UNINEF’7^39^, followed by second-round nested PCR with primers DRIN05^41^ and nefyn05^39^. The conditions for PCR were as follows: in the first PCR, initial denaturation at 94 °C for 2 min followed by 10 cycles of 94 °C for 15 s, 54.5 °C for 30 s, and 68 °C for 6 min; 25 cycles of 94 °C for 15 s, 54.5 °C for 30 s, and 68 °C for 6 min and 20 s; and final extension at 68 °C for 7 min. In the latter cycles, elongation times were extended for 20 s for each cycle. In the second PCR, 62 °C was used as the annealing temperature. For a negative control PCR, sterilized distilled water was added instead of DNA samples.

### Sequence data

Based on the results of an HIV molecular epidemiological survey conducted between 2013 and 2020 in several regions in Indonesia, we obtained 20 new nearly full genome sequences of CRF01_AE [corresponding to nucleotides 790–10,139 of the HIV-1 reference strain, HXB2 (GenBank accession no. K03455)] amplified from proviral DNA with IDs (with the sequence length), MN34 (8428 bp), MN44 (8467 bp), MER UM6 (8,414 bp), HIP6 (8492 bp), HIP10 (8414 bp), CEN5 (8485 bp), PB3 (8468 bp), PB15 (8452 bp), PB16 (8449 bp), PB24 (8446 bp), IDU10 (8462 bp), PJ28 (8461 bp), PJ68 (8410 bp), SBY2-19 (8309 bp), SM44 (8208 bp), BR2 (8,474 bp), BR4 (8554 bp), SS40 (8232 bp), H41 (8355 bp), and H44 (8293 bp). The sequence data obtained were assembled using Genetyx 10 software (Genetyx, Tokyo, Japan). Potential contamination had been evaluated by BLAST search, and no contamination was confirmed for newly amplified viral genomic fragments. The nucleotide sequences of the near full-length HIV-1 genomes have been registered in the GenBank database under accession numbers OQ357234–OQ357253. Using HIV BLAST, we identified closely related CRF01_AE sequences in the HIV-1 database. Sequence quality was analyzed using the Quality Control tool on the LANL HIV Sequence Database (http://www.hiv.lanl.gov), whereas the detection of HIV-1 recombinant or genotype analysis of all sequences was confirmed using the Recombinant Identification Program (https://www.hiv.lanl.gov/content/sequence/RIP/RIP.html) and jumping profile Hidden Markov Model (jpHMM) (http://jphmm.gobics.de). An initial alignment of all 20 sequences was performed using Gene Cutter from the LANL site and then manually adjusted in BioEdit v7.0.9.0.

### Phylogenetic tree

We reconstructed the phylogenetic relationship to confirm possible links between global sequences and Indonesian CRF01_AE clusters. We retrieved 913 CRF01_AE sequences, the majority of which were sampled between 1986 and 2018, from the HIV sequence database at Los Alamos. The accession numbers of downloaded sequence data are shown in the Supplementary Table S2. These constituted the primary geographical spread of CRF01_AE viruses: China (including Hong Kong), Japan, Laos, Thailand, Vietnam, the Philippines, and others (Myanmar, Iran, Central African Republic, Afghanistan, USA, Sweden, Cameroon, Great Britain, Belgium, Slovenia, Zambia, India, and South Africa), to fix the topology of the phylogenetic tree. The phylogenetic trees were rooted using an outlier group containing three subtype C strains: AB485647.1989.ZAM.ZAM18, AB023804.1993.IN.IN101, and U46016.1.ET.1986. Genome sequences were aligned using the multiple alignment using fast fourier transform (MAFFT) program with FFT-NS-i iterative refinement methods^42^. Using the maximum likelihood method in IQ-TREE and the nucleotide substitution model general time reversible (GTR) + F + R10^43^, a phylogenetic tree was constructed. The model was selected as the best fit based on the Bayesian information criterion using the ModelFinder option. To evaluate the robustness of the inferred phylogeny, we performed an approximate likelihood ratio test (aLRT) with 1,000 replicates. Monophyletic groups with aLRT support of ≥ 85% were considered clades. The final tree was visualized using ggtree (v3.4.4) in R^44^. Additionally, the pairwise genetic distance was calculated using IQ-TREE.

### MCC tree

Bayesian phylogenetic inferences were performed for clades containing specimens from Indonesia using BEAST v.1.8.4^45^. The list of specimens used in this method can be found in Supplementary Table S3. We selected eight CRF01_AE specimens from the Central African Republic and Cameroon as the outgroup with the following accession numbers: KP718930, MN116207, MN116208, MN116209, MN116212, AF197340, AF197341, and U51188. The BEAUti module was used to specify the evolutionary model and generate BEAST files. We created and compared eight combinations of substitution, clock, and tree prior models, including Hasegawa-Kishino-Yano (HKY) and GTR (substitution model), relaxed lognormal and strict (clock model), and constant size and Bayesian skyline (tree prior). BEAST was used to simulate a Markov Chain for 10,000,000 generations, with samples taken every 1000 generations. The effective sample size (ESS > 200) and convergence of the posterior distribution were analyzed with Tracer v1.7.2^46^. Based on their superior convergence and ESS values, HKY, strict, and constant-size models were selected for our dataset. Tree Annotator was used to generate the MCC after discarding the 10% burn-in. Meanwhile, the R tool ggtree was used to generate a visual representation of the tree along with its metadata^44^. The visualizations of the MCC tree on geographic maps were performed using SpreaD3^47^.

### Ethical approval

Ethical clearance was obtained from the Institutional Ethics Committees of Airlangga University (approval number: 189/HRECC.FODM/IV/2022) and Kobe University Graduate School of Medicine (approval number: 784). Written informed consent was obtained from all study participants from whom peripheral blood samples were collected. All experiments were performed in accordance with the relevant guidelines and regulations, including these for handling pathogenic microorganisms and for good laboratory practice, of the above listed institutions.

### Supplementary Information

Supplementary Table 1.

Supplementary Table 2.

Supplementary Table 3.

## Data Availability

All necessary data generated or analyzed during the present study are included in this published article and its Supplementary Information files.
